# The role of reactive oxygen species in the immunity induced by nano-pulse stimulation

**DOI:** 10.1038/s41598-021-03342-4

**Published:** 2021-12-09

**Authors:** Siqi Guo, Niculina I. Burcus, Megan Scott, Yu Jing, Iurii Semenov

**Affiliations:** Frank Reidy Research Center for Bioelectrics, Old Dominion University, Norfolk, VA 23508 USA

**Keywords:** Cancer, Immunology

## Abstract

Reactive oxygen species (ROS) are byproducts of tumor cells treated with Nano-Pulse Stimulation (NPS). Recently, ROS have been suggested as a contributing factor in immunogenic cell death and T cell-mediated immunity. This research further investigated the role of NPS induced ROS in antitumor immunity. ROS production in 4T1-luc breast cancer cells was characterized using three detection reagents, namely, Amplex Red, MitoSox Red, and Dihydroethidium. The efficiency of ROS quenching was evaluated in the presence or absence of ROS scavengers and/or antioxidants. The immunogenicity of NPS treated tumor cells was assessed by ex vivo dendritic cell activation, in vivo vaccination assay and in situ vaccination with NPS tumor ablation. We found that NPS treatment enhanced the immunogenicity of 4T1-luc mouse mammary tumor, resulted in a potent in situ vaccination protection and induced long-term T cell immunity. ROS production derived from NPS treated breast cancer cells was an electric pulse dose-dependent phenomenon. Noticeably, the dynamic pattern of hydrogen peroxide production was different from that of superoxide production. Interestingly, regardless of NPS treatment, different ROS scavengers could either block or promote ROS production and stimulate or inhibit tumor cell growth. The activation of dendritic cells was not influenced by blocking ROS generation. The results from in vivo vaccination with NPS treated cancer cells suggests that ROS generation was not a prerequisite for immune protection.

## Introduction

Nano-Pulse Stimulation (NPS) is an approach where cells are treated by nanosecond range electric pulses with short rise and fall times and high electric field strength (10–68 kV/cm), also referred to as nanosecond pulsed electric fields or nanosecond electric pulses^1,2^ in other biological processes. NPS has been demonstrated to be an effective tumor ablation method for various types of cancer in animal models, such as mouse melanoma^3,4^ breast cancer^5^, pancreatic cancer^6^, xenograft human breast cancer^7^, xenograft human pancreatic cancer^3^, and mouse and rat hepatocellular carcinoma^8^. A small clinical trial shows NPS treatment alone is sufficient to completely ablate basal cell carcinoma^9^. In addition to local tumor ablation, a vaccine-like effect has also been reported by several groups^8,10^ including ours^5^. While the exact mechanisms behind a vaccine-like immunogenic cell death [ICD] induced by NPS have not been clearly depicted, they are being studied^5,10,11^.

NPS was suggested as a novel type of ICD inducer^11^. Immune response and vaccine-like protection elicited by NPS treatment has been observed in multiple cancer models, such as rat hepatocellular carcinoma^8^, mouse melanoma^10,12^, mammary cancer^5^, lymphoma, colorectal carcinoma^13^ and pancreatic cancer^6,14^. We previously demonstrated that NPS treated 4T1 cells exhibited Calreticulin [CRT] exposure on the cell surface, released both ATP and HMGB1^5^, and activated dendritic cells in vitro*,* as evidenced by significant upregulation of co-stimulatory molecules CD40, CD86, and MHC-II. These hallmarks of ICD^15–19^ were reported in other cancer cells treated with NPS by Nuccitelli’s^11^ and Muratori’s^13^ groups as well. Taken together, substantial evidence supports NPS is an authentic ICD inducer. However, its underlying mechanisms are not fully clarified.

ICD is a common name for cell death that leads to immune responses^18,20,21^. Reactive oxygen species [ROS] have been demonstrated to play an essential role in the ICD induced by Hypericin based photodynamic therapy as a type II ICD inducer^22,23^. ROS generation and the associated endoplasmic reticulum stress occur in the process of cell death induced by many type I ICD inducers as well^24^. Since ROS generation is mainly a collateral effect of these ICD inducers, the importance of ROS itself as a type I ICD inducer is not clear. ROS signaling pathways have been proven necessary for cell survival, play important roles in cancer development and cell death signaling^25,26^, and are also capable of inducing various types of death pathways^27^. ROS were reported to trigger ICD and consequently result in antitumor immune response^28^. On the contrary, ROS were demonstrated to mediate immune suppression as well^29,30^. Interestingly, ROS release has been reported by two groups in cells treated with NPS^31,32^. Currently, there has been no reported research defining the role of ROS in NPS induced immunity. The determination of immunomodulation effects of ROS in NPS cancer treatment may provide an enhancement approach for the NPS-induced antitumor immunity. In this study we assessed effects of ROS on 4T1 breast cancer cells treated with NPS and determined their role in the immunity resulting from NPS treatment.

## Results

### NPS treatment enhances tumor immunogenicity, results in potent in situ vaccination protection and elicits T cell memory

**Figure 1:**
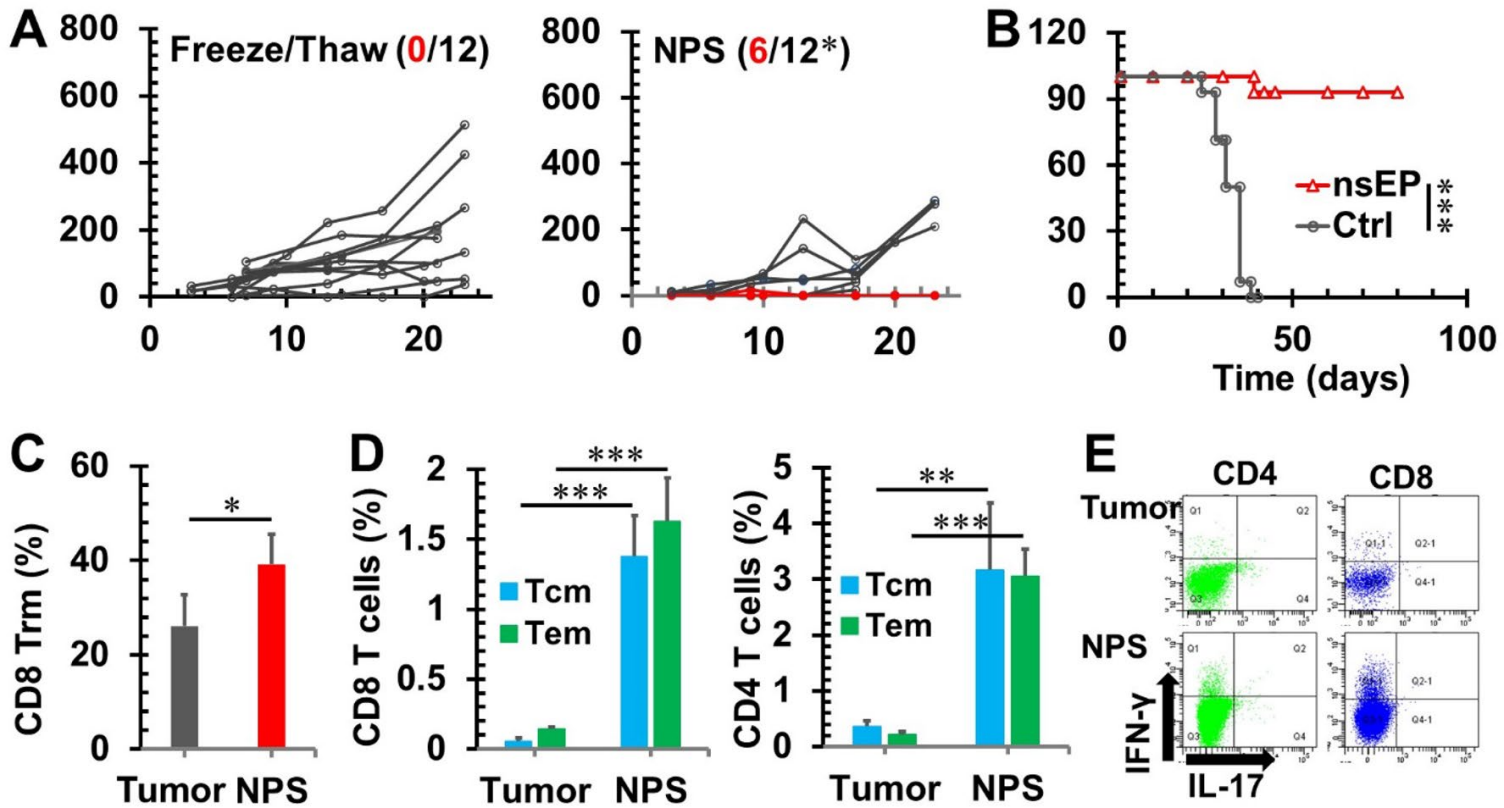
NPS treatment induces antitumor immunity and long-term memory T cell responses. (**A**) 4T1-luc tumor growth curves in mice immunized with 2 × 10^6^ 4T1-luc cells treated with 3-cycles of freeze/thaw, or NPS (60 ns, 50 kV/cm, 1 Hz and 120 pulses). The number of tumor free (red) vs total mice are indicated. (**B**) Survival curves of animals after the secondary tumor challenge. Mice with orthotopic 4T1-luc breast tumors (6–8 mm) were treated with NPS (100 ns, 50 kV/cm, 3 Hz and 1000 pulses). Animals with tumor free over 7 weeks were challenged orthotopically in a different mammary fat pad with 0.5 × 10^6^ live 4T1-luc tumor cells. **Ctrl**: naïve mice without prior NPS treatment (n = 14). **NPS**: tumor free mice after NPS treatment (n = 14). (**C**) The frequency of tissue resident memory CD8^+^ T (Trm) cells in draining lymph nodes. **Tumor:** control animals without NPS treatment (n = 5). **NPS**: 9 days after NPS treatment (n = 5). (**D**) The frequencies of effector (Tem) and central (Tcm) memory T cells in spleen. (**E**) Representative flow plots of intracellular cytokine staining for IFN-γ and IL-17 in T cells from splenocytes cocultured with anti-CD3 for 6 h. For both (**D**, **E**), **Tumor:** control animals without NPS were euthanized at day 35 after tumor initiation (n = 3). **NPS**: animals with tumor free over 3 months after NPS treatment were euthanized (n = 5). *p < 0.05. **p < 0.01. ***p < 0.001. (Chi-Square for A, LogRank for B and *t* test for **C**, **D**, respectively)**.**

Previously, we reported that 4T1-luc cells treated with NPS released surrogate biomarkers of ICD including calreticulin, ATP and high mobility group protein B, and activated DCs in vitro^5^. To further validate if NPS treatment can enhance the immunogenicity of 4T1-luc breast cancer, in vivo vaccination assays were carried out. As shown in Fig. 1A, 4T1-luc cells are poorly immunogenic. All mice immunized with tumor lysate prepared by 3 cycles of freeze/thaw grew tumors, hence resulted in no protection (0% or 0/12) against live tumor challenge. On the contrary, half of mice (50% or 6/12) immunized with NPS-treated 4T1-luc cells rejected live tumor challenge. Therefore, our in vivo vaccination assays indicate NPS is a bona fide ICD inducer.

To assess if NPS treatment in vivo can improve the immunogenicity of cancer, hence turn the tumor itself into vaccine (in situ vaccination), we first treated 4T1-luc tumors with NPS then animals with complete tumor regression seven weeks after NPS treatment were challenged again with the same type of live cancer cells, but at a different location. A potent rejection of tumor growth was shown in Fig. 1B. In contrast to that none of the (0% or 0/14) naïve mice were protected, 93% (13/14) of mice having been treated with NPS were protected from the secondary tumor challenge. This phenomenon of in situ vaccination protection has also been demonstrated in a rat hepatocellular cancer model^8^ and a mouse pancreatic cancer model^14^ treated with NPS.

The mechanisms of NPS-induced potent in situ vaccination protection are unknown. We hypothesize that T cell immunity plays a critical role in the in situ vaccination protection. To test our hypothesis, we examined both early and long-term immune responses. Tissue-resident memory CD8 T cells were significantly increased in draining lymph nodes 9 days after NPS treatment (Fig. 1C). In long-term, central memory and effector memory CD4/8 T cells were remarkedly expanded 9–25 folds in tumor-free mice after NPS treatment in comparison to tumor-bearing animals (Fig. 1D). Furthermore, intracellular cytokine staining showed a prominent population of IFN-γ producing CD4 and CD8 T cells in the spleen of NPS treated mice (Fig. 1E). These analyses suggest T cell immunity is involved in the NPS-induced in situ vaccination protection.

However, major molecular events occurring in tumor cells after NPS treatment that are responsible for DC activation and T cell priming are unexplored. To investigate whether ROS are involved in this process, next we evaluate ROS production from tumor cells and defined their role in the NPS induced ICD.

### ROS production derived from NPS treated 4T1-luc cells is a dose-dependent phenomenon

**Figure 2:**
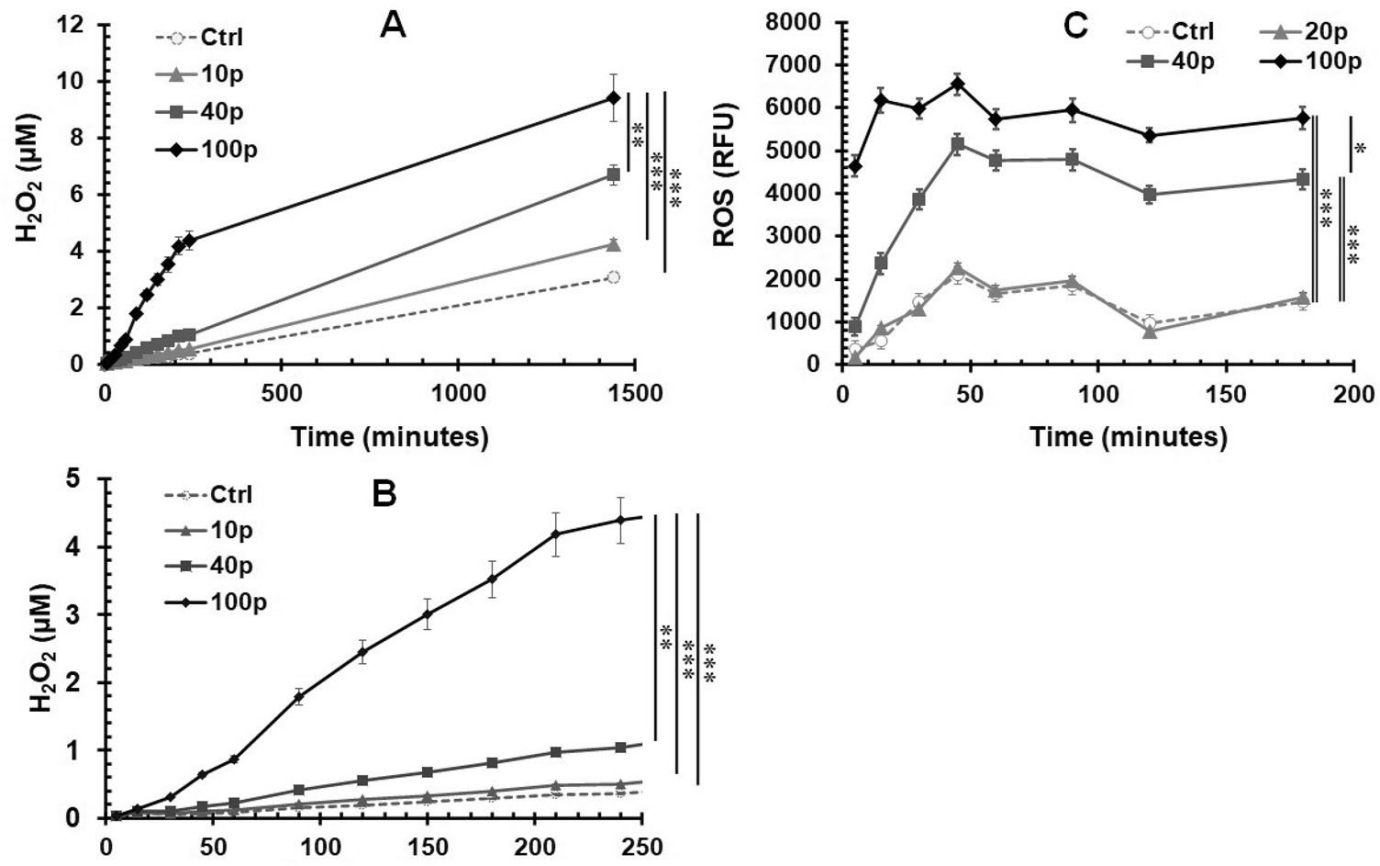
ROS production in 4T1-luc cells treated with NPS. 4T1-luc breast cancer cells were treated with NPS (60 ns, 50 kV/cm, 1 Hz with various pulses numbers). (**A**) and (**B**) ROS (H_2_O_2_) was measured by the Amplex Red Kit. (**C**) ROS (Intracellular superoxide) was detected by dihydroethidium (DHE). Ctrl: control cells without NPS treatment. 10p or 20p, 40p and 100p: treated with NPS with 10 or 20, 40 and 100 pulses (n = 8 each treatment). RFU: relative fluorescence unit. Error bars represent standard errors (n = 8). *p < 0.05, **p < 0.01 and ***p < 0.001 by One Way ANOVA. The double lines indicate one groups vs the other two groups with nearly identical results, respectively.

Determining the kinetics of ROS generation by cells remains a challenge because of their highly active nature and inclusion of multiple species of chemically reactive molecules. Hydrogen peroxide H_2_O_2_ is most commonly measured because it is relatively stable and has a longer half-life amongst other ROS molecules^33^. As shown in Fig. 2A,B, H_2_O_2_ levels correlate with the number of nanosecond electric pulses applied. Within 24 h after treatment, H_2_O_2_ levels also show time dependent increases. The concentration of H_2_O_2_, 1–4 μM in 1–4 h after exposure to 100 pulses, that our group obtained, is higher than that reported by Dr. Pakhomov’s group^31^. Considering that even 100 μM H_2_O_2_ does not have toxicity to 4T1 cells^34^, this level of H_2_O_2_ induced by NPS is unlikely to impact cell viability however, importantly, it can serve as an intracellular signaling molecule.

Besides measuring H_2_O_2_, DHE has been often used to measure intracellular superoxide levels^35–37^. The superoxide level increased in an NPS dose-dependent manner within as early as 5 min after NPS treatment. As shown in Fig. 2C, cells treated with the lethal dose (100 pulses) of NPS produced the highest level of superoxide. Regardless of pulse numbers applied, levels of superoxide peaked in 45 min and remained relatively stable for up to at least 3 h. This pattern of superoxide release is very different from that of H_2_O_2,_ showing continuous increases of H_2_O_2_ levels in the first 4 h upon pulse delivery (Fig. 2B).

**Figure 3:**
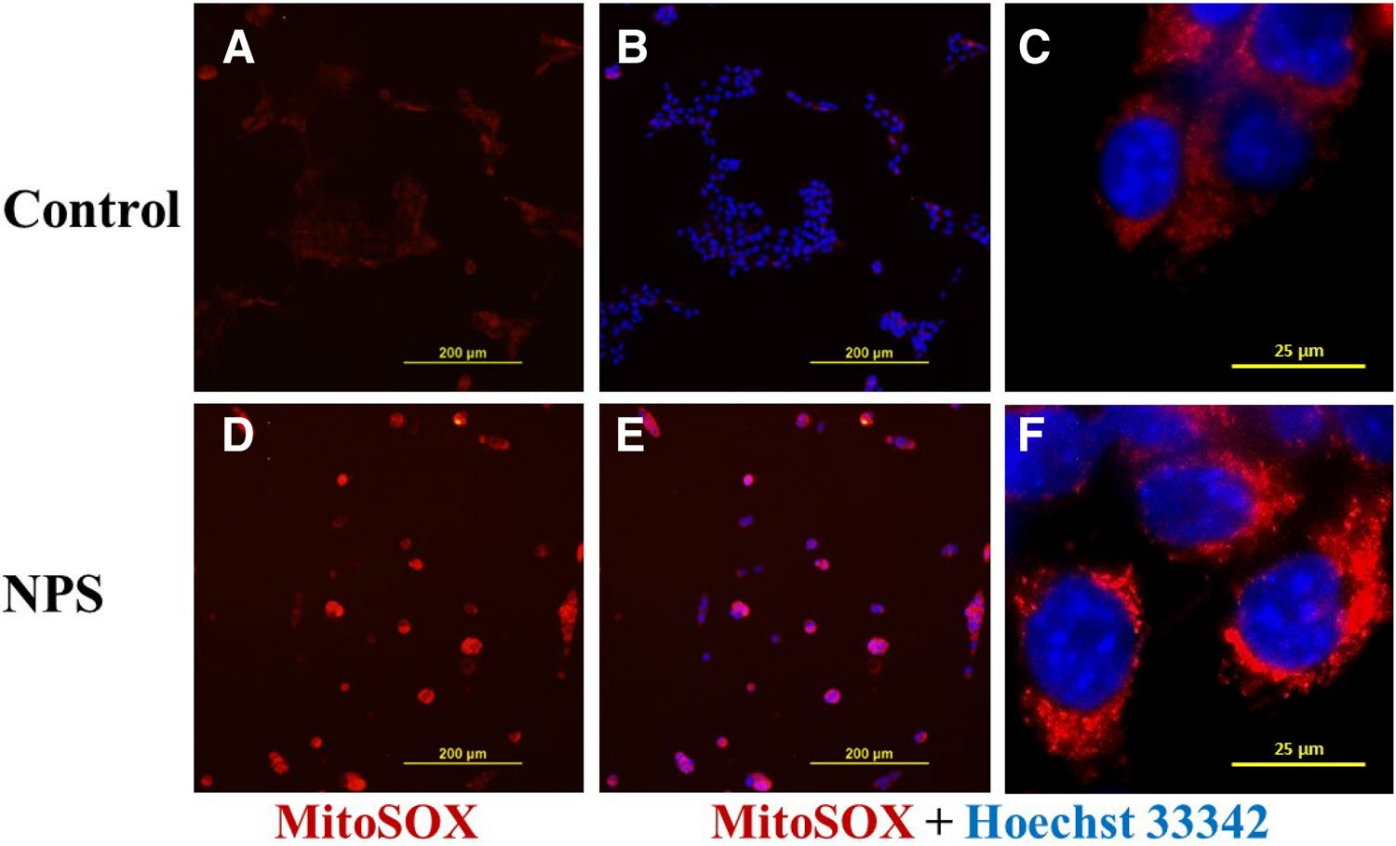
Intracellular ROS increase after NPS treatment. 4T1-luc breast cancer cells were treated with NPS and incubated at 37 °C overnight. After stained with 5 μM MitoSOX red and 0.5 μg/mL Hoechst 33,342 cells were examined under fluorescence microscope. (**A**) and (**D**) images show oxidized MitoSOX red; (**B**, **C**) and (**E**, **F**) images show both nuclear stained with Hoechst 33,342 (blue) and cytoplasm stained with oxidized MitoSOX red (red). Control: cells without NPS treatment. NPS: cells treated with NPS, 60 ns, 50 kV/cm, 1 Hz and 100 pulses. One representative image from three samples with the same treatment was presented.

MitoSox red reagent has been utilized for selective detection of superoxide changes in the mitochondria^38^. A significant increase of red fluorescence level has been observed in cells treated with 100 pulses after overnight incubation at 37 °C (Fig. 3A–F). In contrast, control cells showed a low level of superoxide. Noticeably, there is also a large difference between control cells and NPS treated cells in terms of cell morphology. Control cells attach well, with sheets of cells and even the size of the nucleus, whereas a high heterogeneity of NPS treated cells in size, shape, and nuclear morphology suggests that they are undergoing the dying process and are at different stages of this process.

### ROS scavengers can block or promote ROS production induced by NPS depending on the concentration and type.

**Figure 4:**
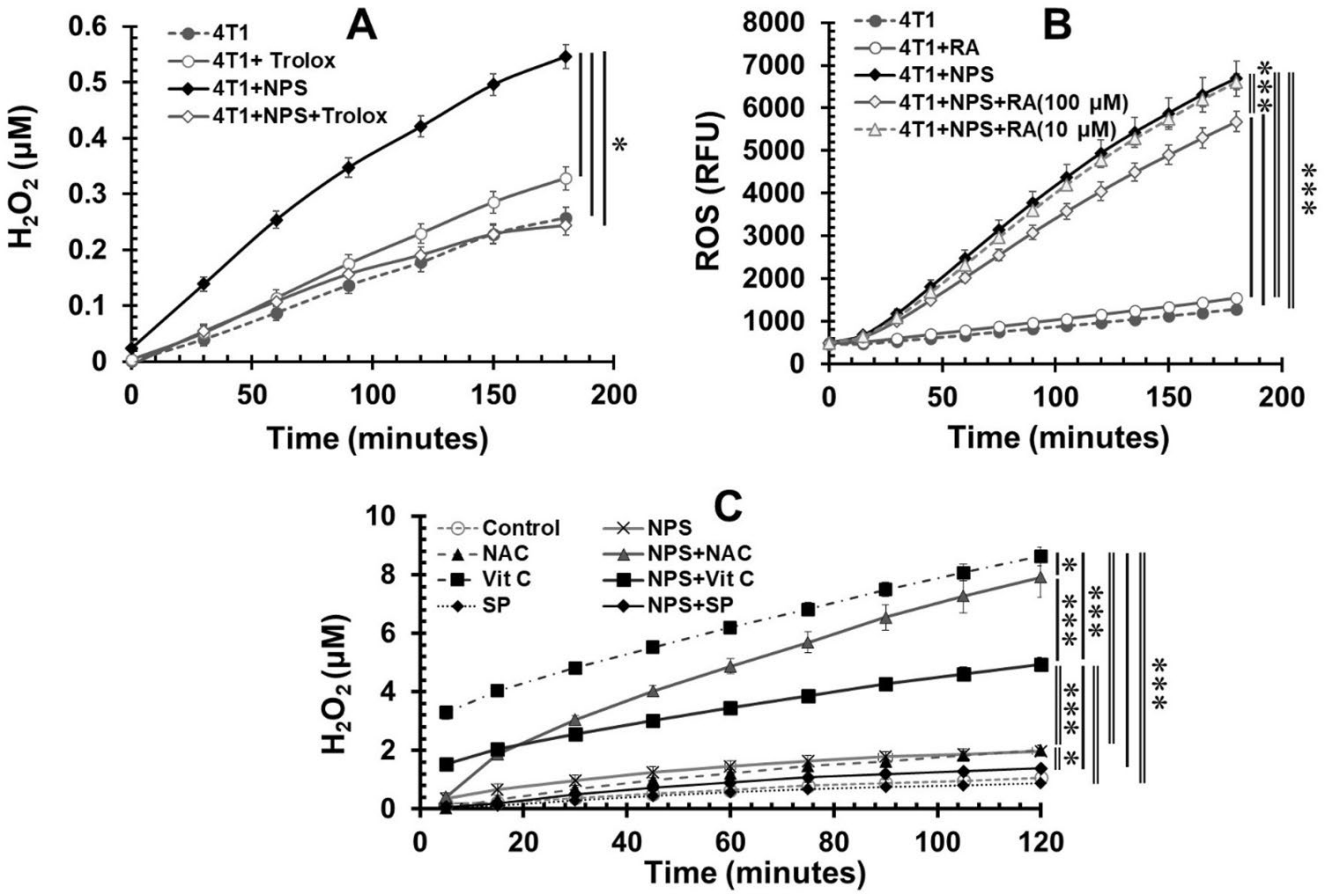
Effect of ROS scavengers/antioxidants on the ROS production induced by NPS. 4T1-luc cells were pretreated with various potential ROS blockers then pulsed with NPS (60 ns, 50 kV/cm, 1 Hz and 100 pulses). The kinetics of ROS (H_2_O_2_) was examined by Amplex Red Hydrogen Peroxide/Peroxidase Assay Kit. (**A**). Effect of Trolox on the H_2_O_2_ generation. (**B**) Effect of Rosmarinic acid (RA) on the on the H_2_O_2_ generation. (**C**) Effect of N-acetyl cysteine (NAC), Vitamin C or SP on the H_2_O_2_ generation. 4T1: 4T1-luc cells without NPS treatment as control. NPS: treated with NPS alone. Trolox, RA, NAC, Vit C or SP: pretreated with Trolox (1 mM), RA (100 μM or 10 μM), NAC (3 mM), Vitamin C (0.5 mM) or sodium pyruvate (10 mM). Error bars represent standard errors (n = 8). *p < 0.05 and ***p < 0.001 by One Way ANOVA. The double lines indicate one group vs the other two groups with nearly identical results, respectively.

There are many ROS scavengers and antioxidants available to quench or block ROS production. We screened several commercially available ROS scavengers to determine which are the best at reducing or blocking ROS production with minimal impact on cell viability. Trolox was reported to effectively block ROS production induced by NPS treatment in human pancreatic cancer cells BxPC-3^32^. Our results showed that in 4T1-luc cells Trolox (1 mM) completely blocked H_2_O_2_ production induced by NPS but had no effect on spontaneous H_2_O_2_ generation (Fig. 4A). However, RA, which has been reported to have ROS scavenger activity^39^, only slightly reduced the H_2_O_2_ production induced by NPS. Pre-incubation with RA (100 μM) decreased the concentration of H_2_O_2_ in 4T1-luc cells (measured 1 h after NPS exposure) by only 18.5%. No significant changes occurred with RA applied at a lower concentration (10 μM) (Fig. 4B). Other ROS scavengers, Vitamin C^40^, NAC^41^, and sodium pyruvate^42^ have been reported to reduce ROS production and to protect cells from the ROS-related toxicity. Surprisingly, both Vitamin C (0.5 mM) and NAC (3 mM) significantly elevated H_2_O_2_ production induced by NPS treatment. Overall, cells pre-incubated with Vitamin C produced much more H_2_O_2_ than control cells (Fig. 4C). In contrast, cells pre-incubated with NAC showed the same level of H_2_O_2_ as control cells at the start but gradually generated more H_2_O_2_ over time (Fig. 4C). Sodium pyruvate (10 mM) was able to reduce H_2_O_2_ production induced by NPS treatment close to control levels and did not enhance spontaneous H_2_O_2_ generation.

### ROS scavengers can impact cell viability at low NPS doses but have lesser effects at lethal NPS doses.

**Figure 5:**
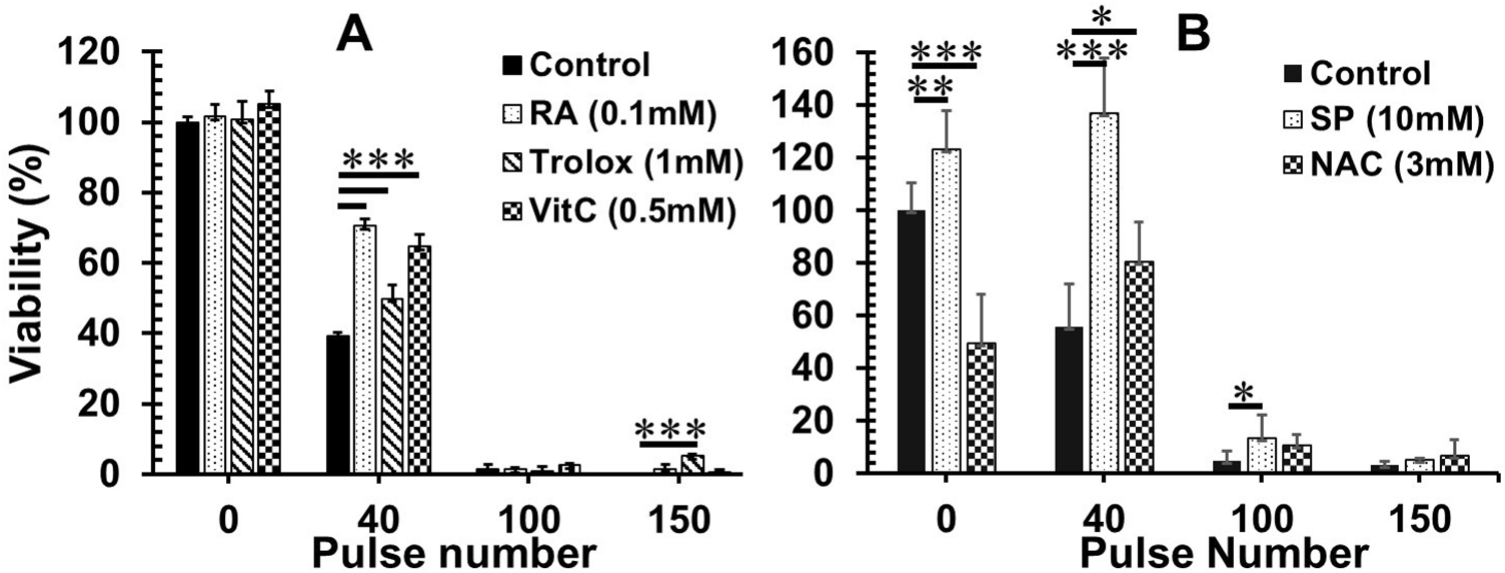
Effect of ROS blockers on the viability of cells treated with NPS. 4T1-luc cells were preincubated with various ROS blockers for 30 min at 37 °C then 100 μL, cells at a concentration of 5 × 10^6^ cells/mL in a 0.1 cm-gap cuvette were treated with NPS (60 ns, 50 kV/cm, 1 Hz and pulse numbers indicated). Cell viability was measured by WST assays after an 18-h incubation at 37 °C and 5% CO_2_. (**A**) Effect of rosmarinic acid (RA), Trolox and Vitamin C on the cell viability. Control: cells treated with NPS alone; RA (0.1 mM), Trolox (1 mM) or Vit C (0.5 mM): preincubated with RA at 0.1 mM, Trolox at 1 mM or Vitamin C at 0.5 mM then treated with NPS. (**B**) Effect of SP and NAC on the cell viability. Control: cells treated with NPS alone; SP (10 mM) or NAC (3 mM): cells preincubated with sodium pyruvate at 10 mM or N-acetyl cysteine at 3 mM then treated with NPS. Error bars represent standard deviations (n = 8). *p < 0.05, **p < 0.01 and ***p < 0.001 by One Way ANOVA.

Next, we studied if ROS scavengers could impact cancer cell viability following NPS treatment. As shown in Fig. 4, most antioxidants do not affect 4T1-luc cell growth (Fig. 5A). On the other hand, sodium pyruvate (SP), at 10 mM, stimulated growth of 4T1-luc cells whereas NAC at 3 mM showed toxicity to 4T1-luc cells (Fig. 5B). The viability of 4T1-luc cells increased by 23% with the addition of 10 mM sodium pyruvate and decreased 51% with the addition of 3 mM NAC (Fig. 5B). In 4T1-luc cells antioxidants showed various levels of protection at low doses of NPS but were less potent or lost their effectiveness at high doses of NPS. For instance, viability of 4T1-luc cells treated with 40 pulses increased by 80%, 27%, 65%, 146% and 44% with the addition of RA 0.1 mM, Trolox 1 mM, Vitamin C 0.5 mM (Fig. 5A), sodium pyruvate 10 mM, and NAC 3 mM, respectively (Fig. 5B). Nevertheless, when 4T1-luc cells were treated with 100 or 150 pulses which resulted in 95–100% of cell death, only Trolox and sodium pyruvate exhibited some extent of protection. This falls in-line with their potency of blockage of ROS production generated by high dose of NPS (Fig. 4A,C).

### ROS scavengers do not block ex vivo dendritic cell activation by NPS-treated 4T1-luc cells

**Figure 6:**
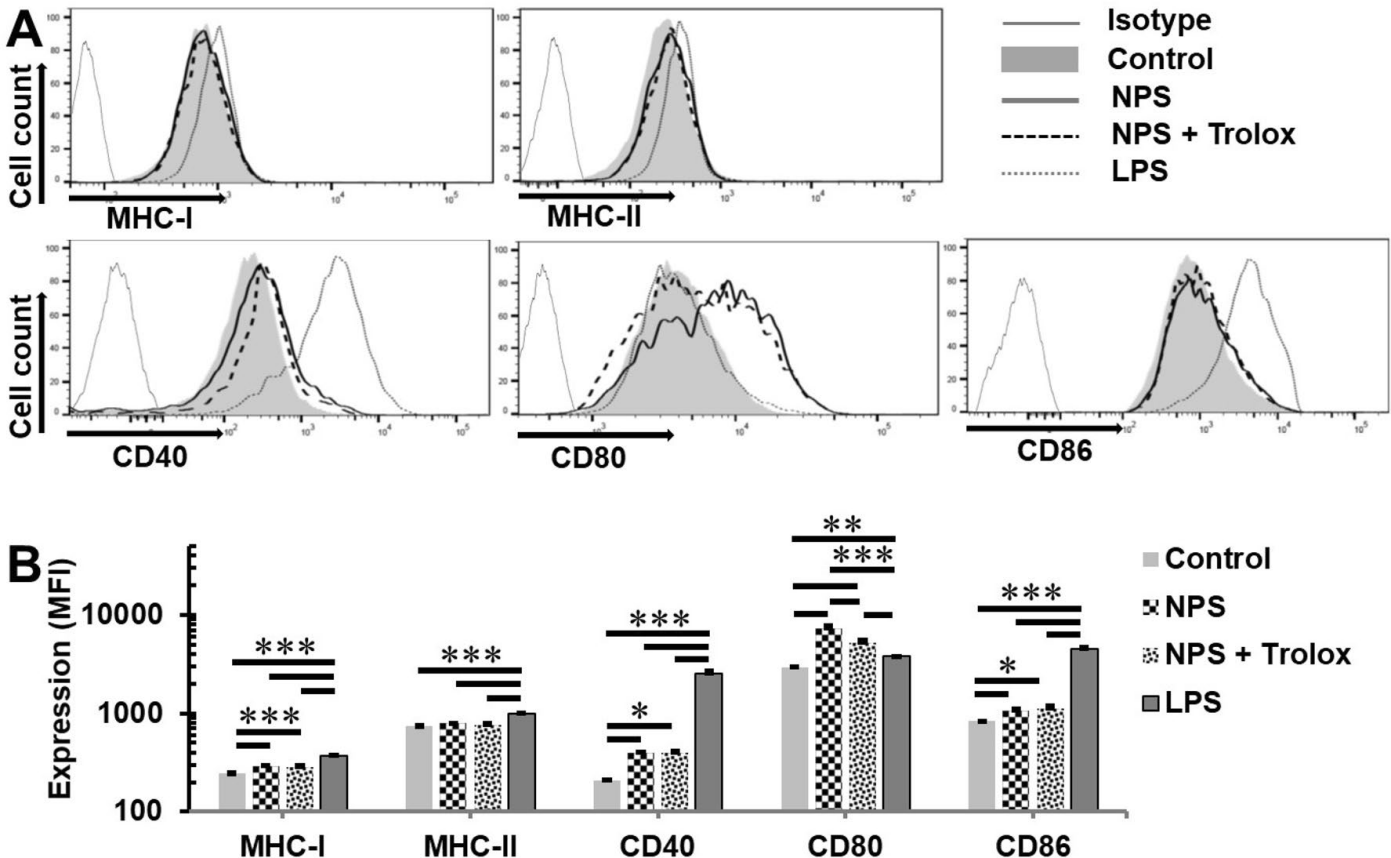
Effect of Trolox on cell surface markers of dendritic cells stimulated with NPS treated breast cancer cells. Bone marrow derived dendritic cells (BMDCs) were incubated with media only (Control), lipopolysaccharide (LPS) 5 μg/mL, the NPS-treated 4T1 cells (NPS) or the NPS treated 4T1-luc cells with Trolox 1 mM (NPS + Trolox). Two day later, cells were collected for the analysis of surface activation markers by flow cytometry. (**A**) Results of one representative histogram for each treatment from two independent experiments (n = 3 and 4, respectively) were shown here. (**B**) Expression of cell surface molecules of dendritic cells was measured by mean fluorescent intensity (MFI). Error bars represent standard deviations (n = 4). *p < 0.05, **p < 0.01 and ***p < 0.001 by One Way ANOVA.

Activation or maturation of dendritic cells is a prerequisite of immune response induction. Here we examined five cell surface molecules commonly used as biomarkers for dendritic cell activation. Consistent with our previous report, incubation of dendritic cells with NPS treated 4T1-luc cells upregulates MHC-I, CD40, CD80 and CD86 markers (Fig. 6A,B). However, 1 mM Trolox, which was shown to effectively block H_2_O_2_ generation in 4T1 cells treated with NPS (Fig. 3A), did not change the expression of these activation markers except the partial inhibition of CD80 upregulation by NPS. In contrast, dendritic cells stimulated with LPS expressed the highest level of MHC-I, MHC-II, CD40, and CD86.

### Blockade of ROS production does not diminish immunogenicity of NPS treated 4T1-luc cells

**Figure 7:**
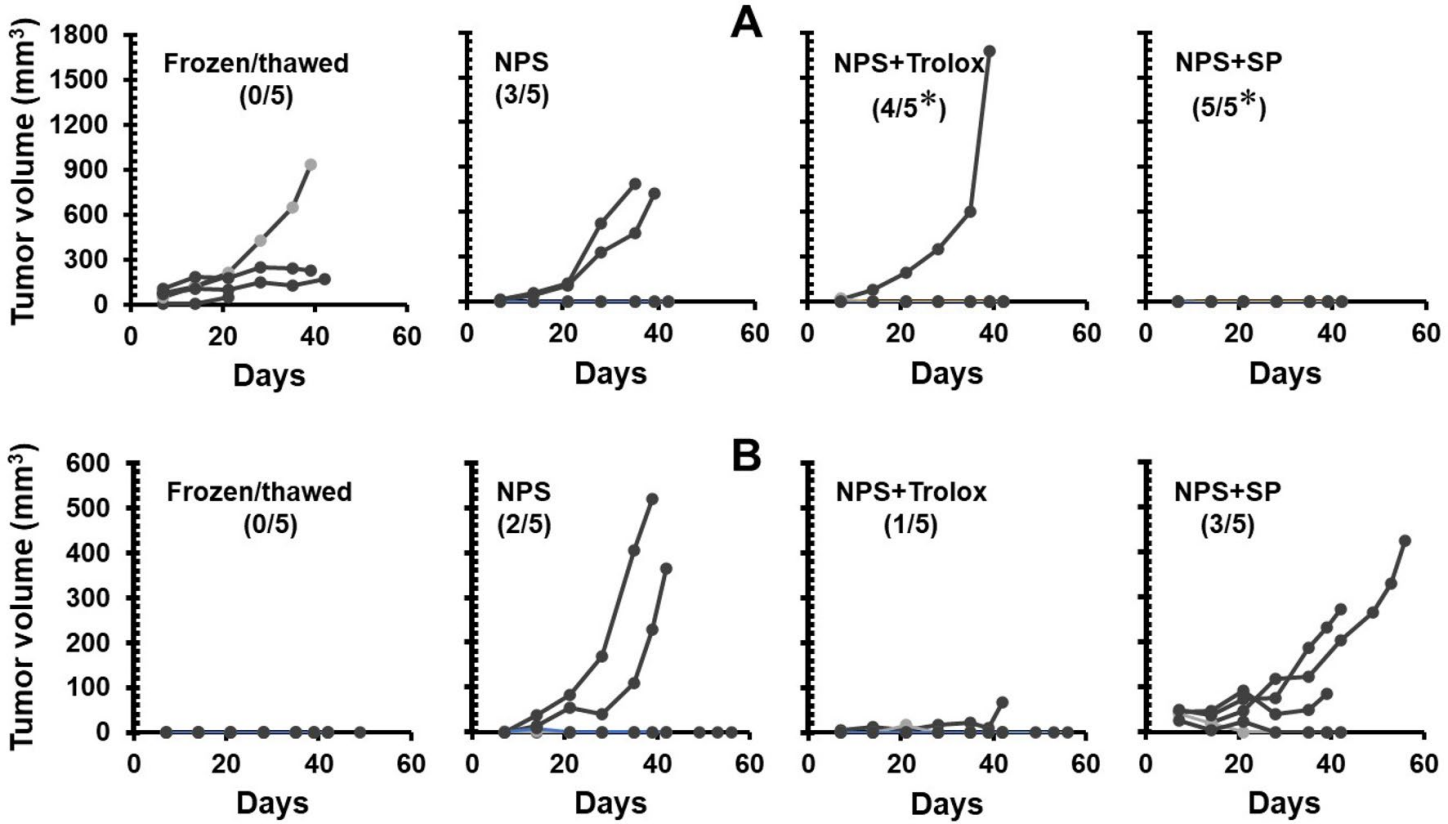
Effect of ROS blockers on the NPS induced immune protection. Female Balb/c mice were immunized subcutaneously with 4T1-luc tumor lysate (Frozen/Thawed), NPS treated cells (NPS), NPS treated cells with preincubation of Trolox 1 mM (NPS + Trolox) or sodium pyruvate 10 mM (NPS + SP). Ten days after immunization animals (n = 5 for each group) were challenged with intra-mammary live 4T1-luc cells. (**A**) Growth curves of challenge intra-mammary tumors. Numbers indicate animals with challenge tumor rejection above total animals. (**B**) Growth curves of subcutaneous tumors at the immunization sites. Due to large variations in tumor volumes, tumor growth curves in each group are shown as individual mice. Each curve represents tumor growth in one mouse. The rates of vaccine protection (tumor rejection) or tumor growth are compared among treatment groups. Numbers indicate animals with subcutaneous tumor growth above total animals. *p < 0.05 by Chi square test.

To examine if immune protection induced by NPS is affected by ROS blockers, in vivo vaccination assays were carried out. Since our in vitro results showed Trolox and sodium pyruvate were effective ROS blockers for the abolition or reduction of H_2_O_2_ induced by a lethal dose of NPS, the next step was to test if the blockade or reduction of ROS could eliminate or diminish the immune protection of NPS treated 4T1-luc cells. As shown in Fig. 7A, immunization with NPS treated cells protected 60% (3/5) of animals from live tumor challenge whereas tumor lysed by freeze/thaw as the antigen source did not result in any animal protection (0/5). Both the reduction and abolition of ROS did not decrease protection rates, which were 80% or 100%, respectively, to NPS treated cells with the addition Trolox or sodium pyruvate. The rate of protection by NPS with the addition of Trolox or SP was actually higher than that of NPS alone treated 4T1-luc cells. Although such a rate increase did not reach statistical significance, the results suggest ROS production or H_2_O_2_ levels do not play a role in the immunity induced by NPS for the 4T1-luc breast cancer model. One issue with this vaccination approach was that tumor could grow at the vaccination site. As shown in Fig. 7B, no mice immunized with frozen/thawed cells grew tumor at the vaccination site, however, some of the mice immunized with NPS treated cells did grow tumor at the vaccination site. There were 40% (2/5), 20% (1/5), or 60% (3/5) of mice growing tumor after vaccination with 4T1-luc cells treated with NPS, with additional Trolox, or with sodium pyruvate, respectively. Based on our viability assays, the dose of adopted NPS (100 pulses 60 ns, 50 kV/cm at 1 Hz) could kill 95–99% of 4T1-luc cells. However, this result indicated that only 30,000–150,000 (1–5% out of 3 × 10^6^) surviving 4T1-luc cells were sufficient to establish a tumor. Viability assays could also explain why more tumors grew at the vaccination site in animals immunized with NPS and sodium pyruvate treated cells. Mainly because sodium pyruvate increased the viability of cells treated with a lethal dose of NPS (Fig. 5B).

## Discussion

We validated NPS is an authentic ICD inducer and induced a potent in situ vaccination protection in a poorly immunogenic mouse breast cancer model. Significant increase of memory T cells were associated with long-term immune protection against live tumor challenge. We observed dose-dependent ROS production in 4T1-luc breast cancer cells treated with NPS. We found that certain ROS scavengers/antioxidants did block or reduce ROS production while other scavengers/antioxidants did not reduce but, in fact, even promoted ROS production following NPS treatment. ROS scavengers Trolox and RA showed protective effects when cells were treated with low doses of NPS but were less effective in cells treated with lethal doses of NPS. Nevertheless, we were able to identify two ROS scavengers that could effectively block ROS production after NPS treatment in 4T1-luc breast cancer cells. Importantly, the blockade of ROS by these two ROS scavengers neither altered the activation of dendritic cells nor reduced the vaccine effect of NPS treated cells. Therefore, ROS production is unlikely the decisive factor of NPS induced vaccine effects.

Our data together with our publication^5^ suggest NPS is an ICD inducer for breast cancer. Rossi et al. reported that NPS can enhance the immunogenicity of mouse MCA205 fibrosarcoma and CT-26 colon cancer. Vaccination with NPS treated EL-4 lymphoma and CT-26 cancer cells also protected 50% and 78% of animals against live tumor challenge, respectively^13^. Importantly, NPS tumor ablation elicits a strong in situ vaccination protection as well. Various rates of in situ vaccination protection were observed in other cancer models treated with NPS. NPS treatment can result in 100% (21/21), 75% (8/12) or 33% (6/18) of animals rejecting the secondary live tumor challenge, respectively, in rat N1S1 hepatocellular^8^, mouse Pan02 pancreatic^14^ or B16 melanoma^12^ models.

Diverse immune outcomes following NPS treatment in different cancer models emphasize the importance of its underlying mechanisms. Previously, we discovered NPS treatment activated antigen presenting cells, dramatically decreased immune suppressive cells both in blood and tumor microenvironment^5,6^. Here we found a significant increase of tissue-resident memory T cells at early time point and a remarkable expansion of effector/central memory T cells in later time-period. Our results indicate T cells are involved in the NPS-induced in situ vaccination protection. Noticeably, Nuccitelli et al. reported CD8 T cells were responsible for the inhibition of secondary tumor growth after primary tumor was ablated with NPS in the rat McA-RH7777 liver cancer model^43^.

Various ROS detecting agents have been adopted to measure ROS in different types of cells. Pakhomov’s group employed 2′,7′-dichlorodihydrofluoresein (H2DCF), DHE, and Amplex Red to measure ROS products in Jurkat (human T-lymphocytes), U937 (human monocytes), and CHO (Chinese hamster ovary) cells^31^. H2DCF has also been used by Nuccitelli’s group to detect intracellular ROS of BxPC-3 [human pancreatic cancer) cells^32^. In our study, Amplex Red and DHE were utilized to examine ROS derived from 4T1 breast cancer cells. Although different cell types were treated with various parameters of NPS in the indicated studies, a proportional increase of ROS to the dose of NPS (the number of pulses) has been observed in all studies. It appears that the release of ROS products is confirmed both intra- and extracellularly. One argument from Dr. Pakhomov’s report is that the source of ROS, especially H_2_O_2_, can be generated from the cell free media, specifically RPMI growth medium without serum and without phenol red, simple salt buffer, and PBS exposed to NPS. In our study, the complete cell culture media; high glucose DMEM with 10% fetal bovine serum, non-essential amino acids and antibiotics, was used primarily for two reasons. One being that the measurement of ROS is carried out periodically for up to 24 h so complete media is used for the survival of control cells and to avoid nutritional shortage stress. The second reason being that complete media most closely mimics the in vivo tumor ablation setting which has been reported to result in vaccine-like effects^5,8^. Despite a concern of pro-oxidant effects of free iron in the DMEM^44^, the H_2_O_2_ measurement is reported as not dependent on DMEM with or without serum^45^. In addition, a continuously increasing but not a bursting pattern of H_2_O_2_ indicates a gradual generation of ROS from the electric pulse-stressed cells (Figs. 2A, 4A-C) instead from the culturing media. Moreover, the results of MitoSox staining which targets mitochondria specifically suggest mitochondria are one source of ROS (Fig. 3).

Surprisingly, not all antioxidants/scavengers are able to block or reduce H_2_O_2_ generation by NPS. Our results show 4T1-luc cells pretreated with NAC significantly increase the production level of H_2_O_2_ by NPS (Fig. 4C). However, it appears NAC alone has no effect on H_2_O_2_ generation at the beginning of post-NPS treatment but gradually increase H_2_O_2_ generation over time. This indicates the dose (3 mM) adopted here may cause certain cytotoxicity, which is consistent with our viability result (Fig. 5B). The autooxidation of thiols including NAC was proposed by several researchers^46,47^ however, this may explain the increase of H_2_O_2_ with NAC alone but does not explain why NPS elevates H_2_O_2_ production. Another unexpected result is that Vitamin C induces very high levels of H_2_O_2_ (Fig. 4C). In this case, NPS seems to weaken Vitamin C’s effect on ROS induction. In both cases, the exact mechanisms of “autooxidation” of antioxidants and the impact of NPS are unknown. Nevertheless, RA, Trolox, and sodium pyruvate at the applied concentration(s) do not show autooxidation. Their abilities to block ROS induced by NPS are different where Trolox and sodium pyruvate significantly decrease ROS equal or close to the level of endogenous H_2_O_2_ whereas RA exhibits only a partial blocking effect. It is necessary to point out limitations regarding these assays and results. Factors, including doses of the antioxidants and media/buffers used in the reactions have not been optimized, potentially altering the outcome.

Though ROS scavengers and antioxidants are different in terms of their working mechanisms, both groups of substances are widely utilized to prevent cell damage or death from excessive ROS induced by drugs or diseases^39–42,48–50^. Our results appear to support this concept. All five selected ROS scavengers/antioxidants protect 4T1-luc cells from cytotoxic effect of NPS. Noticeably, when there is no NPS treatment, sodium pyruvate can stimulate breast cancer cell growth whereas other scavengers except NAC do not impact cell viability (Fig. 5). NAC results in the reduction of cell viability in our study, which suggests the dose (3 mM) adopted here is toxic to 4T1-luc cells. The phenomenon of growth stimulation of pyruvate has been reported by other group as well^51^. However, the potency of cellular protection varies amongst those scavengers and depends on the dose of NPS as well. RA, Vitamin C, and NAC lose protective effects at lethal doses of NPS whereas Trolox and sodium pyruvate significantly enhance cell viability at the same doses. Although the mechanisms causing this difference are unknown, we speculate it is associated with their ability to reduce ROS because both Trolox and sodium pyruvate show a significant reduction of ROS in cells treated with lethal doses of NPS while others do not. The protective effect of sodium pyruvate also leads to the issue of higher rates of tumor growth at the inoculation site when those treated cells are used for a tumor vaccine (Fig. 7).

Unlike other groups’ studies and suggestions that ROS are associated with immunogenic cell death resulting from chemotherapeutic drugs (anthracyclines^52^) and hypericin-based photodynamic therapy^24,53^, the blockade or diminution of ROS generation from NPS does not reduce either ex vivo dendritic cell activation or in vivo vaccine-like protective effect. On the contrary, the immunization study shows more animals are protected from tumor challenge following vaccination with an additional ROS blocker. Though a small number of animals were used in the studies and the differences do not reach statistical significance, similar trends in both groups of animals with ROS blockers hints to the distinct role of ROS in NPS induced immunity from that in chemotherapy. Another puzzle is the contradiction that there is a separation between vaccine-like effects and tumor growth at the inoculation site. The highest tumor protection rate results from NPS and sodium pyruvate treated tumor cells, which also leads to more tumoral growth at the immunization site. As we mentioned above, increased tumor growth at the inoculation site could be explained by an increase of viability but how additional sodium pyruvate improves the protection remains an unknown. Nevertheless, our data imply that ROS is involved in cell death caused by NPS but is unlikely a decisive factor for the NPS-associated vaccine effect. This supports several groups’^8,10,11^ including ours^5,54^ proposal that NPS is a novel physical ICD inducer.

We are aware of the limitations of this study due to our available resource. Only one pulse duration (60 ns) of NPS was studied in one cancer model. The role of ROS in the cell death mechanism was not defined. Whether the blockage of ROS could change cell death pattern and consequently enhance or reduce the immunogenicity of NPS treated cancer cells was not elucidated either. Therefore, the ROS generation by other NPS parameters in additional cancer types and its association with immunogenicity should be assessed further to determine if our discoveries are general cross various tumor types or tumor-type specific.

In summary, we have demonstrated that 4T1-luc mammary cancer cells treated with NPS in vitro enhance the tumor immunogenicity. NPS tumor ablation leads to a potent in situ vaccination protection and elicits long-term T cell immunity. The increase of both extracellular and intracellular ROS production has been observed after tumor cells were treated with NPS. The release of ROS production correlates to the dose of NPS. Our data supports that mitochondria are one source of ROS generation. To block ROS release from NPS treatment, the dose and type of scavengers should be optimized to avoid toxicity from the scavenger itself and insufficient potency. ROS scavengers partially protect cells from death induced by NPS, but even complete blockage of ROS does not prevent cell death under lethal doses of NPS. This indicates ROS release may contribute to some extent to the cell death resulting from NPS but is not the major contributor for that cell death. When dendritic cells are stimulated by NPS treated cancer cells, the upregulation of their activation markers is not halted by the blockade of ROS (in cancer cells). The immunogenicity of NPS treated cancer cells do not diminish with the reduction or blockage of ROS. Taken together, this study suggests NPS is likely a type I ICD inducer. Currently, we are investigating the NPS-induced cell death mechanism and its correlation with tumor immunogenicity. The potential enhancement of immunogenicity with the blockage of ROS will be explored as well. Our hope is the elucidation of NPS-elicited immune protection could help us further improve the therapeutic efficacy of this novel in situ vaccination approach.

## Materials and methods

### Cell line

4T1-luc murine breast cancer cells were originally provided by Dr. Gary Sahagian at Tufts University and have been maintained in high-glucose DMEM (ATCC 30–2002) (ATCC)-supplemented with 10% fetal bovine serum, non-essential amino acids, and antibiotics (100 units/mL penicillin and 100 μg/mL streptomycin) (three items above from Atlanta Biologicals). 4T1-luc cells, passage numbers between 2 and 7 were thawed for expansion, and cells with passage numbers between 10 and 20 were used in the described ex vivo or in vivo experiments. Cells were tested periodically to ensure no mycoplasma contamination was present.

### Mice and tumor models

Female Balb/c mice (8—10 weeks of age) were purchased from Jackson Laboratory and housed in the ODU animal facility accredited by the AAALAC. 4T1-luc tumor was initiated by an inoculation of 1 × 10^6^ live 4T1-luc cells in the left posterior mammary fat pad in female Balb/C mice. The size of tumor was assessed by digital calipers twice a week. Tumor volume was determined using the following formula: V = πab^2^/6, where (a) is the longest diameter and (b) is the shortest diameter perpendicular to (a). All experimental protocols were approved by Old Dominion University Institutional Biosafety Committee (IBC) and Institutional Animal Care and Use Committee (IACUC). And all experiments were performed in accordance with relevant guidelines and regulations. At the end of the follow-up period or at specified time points described in experimental designs, the euthanasia of mice was carried out by CO_2_ inhalation.

### Reagents and antibodies

The Amplex Red Hydrogen Peroxide/Peroxidase Assay Kit including 3% H_2_O_2_ for the establishment of standard curve (Cat # A-22188), Invitrogen 96-well microplates for fluorescence-based assays (Cat # M33089), dihydroethidium (DHE) (Cat # D1168), Hoechst 33342 (Cat # H3570), and MitoSOX Red mitochondrial superoxide indicator (Cat # M36008) were purchased from Invitrogen. Trolox (Cat # SC-200810) was obtained from Santa Cruz Biotechnology. Lipopolysaccharides (LPS) from *Escherichia coli* O111:B4 (Cat # L4391), Rosmarinic acid (RA) (Cat # 536954-5G), N-acetyl cysteine (NAC) (Cat # A9165), Vitamin C (Cat # A4403), and sodium pyruvate (Cat # P5280) were purchased from Sigma-Aldrich. WST-1 (Cat # 11644807001) for cell viability assays was obtained from Roche Applied Science. Rat anti-mouse CD3 pacific blue (Cat #100334), CD4 FITC (Cat # 100406), CD8 Percp (Cat # 100732), CD44 APC (Cat # 103012), CD62L PE/Cy7 (Cat # 104418), IFN-γ PE (Cat # 505807), IL-17 PE/Cy7 (Cat # 506921), CD103 PE (Cat # 121406), CD86 Pacific blue (Cat # 105021), I-A/I-E FITC (Cat # 107605), CD40 APC (Cat # 124611), anti-mouse CD16/32 (Cat # 156603), hamster anti-mouse CD11c PerCP (Cat # 117326), hamster anti-mouse CD80 PE/Cy7 (Cat # 104734) and mouse anti-mouse H-2Kd/H-2Dd PE (Cat # 114708) were purchased from BioLegend.

### In vitro NPS treatment

In vitro NPS treatment for cancer cells was described in our previous publication^55^. Briefly, a custom-made nanosecond pulse generator was used to generate 60 ns (ns) electric pulses with various pulse frequencies and applied electric fields of interest. The 60 ns pulse generator is a pulse forming line (PFL), constructed out of five 50 Ω cables connected in parallel for a matching load of 10 Ω. The cable length was selected to allow for a pulse to travel round-trip within 60 ns. A 1-mm cuvette loaded with 0.1 mL cell solution was a load for the generator and to match the PFL for a square pulse. A spark gap switch with adjustable distance was used to close the PFL and the switching medium was air for self-breakdown. Because of the stochastic nature of the air breakdown, the pulse voltage varied within ± 10%. 4T1-luc cells, 100 μL at a concentration of 5 × 10^6^ cells/mL in a 0.1 cm-gap cuvette were pulsed with NPS; pulse duration of 60 ns, frequency of 1 Hz, applied electric field of 5 kV (or 50 kV/cm), and pulse number of 10 to 150 dependent on experimental design. The electrical energy per pulse, which was estimated with the pulse power (2.5 × 10^6^ Watts) and duration (60 ns), is 0.15 Joule.

### In vivo NPS treatment and the secondary tumor challenge

In vivo NPS treatment protocol was detailed in our published paper (5). The 100 ns pulse generator is a Blumlein line, and it was constructed with one 50 Ω cable. In this case, the matched load was 100 Ω. In addition to the tissue resistance, a 50 Ω resistor was added in parallel to ensure a square pulse was produced. Mice with tumors (6–8 mm) were randomly grouped according to tumor volume and treated with NPS (100 ns, 50 kV/cm, 3 Hz and 1000 pulses). Animals with tumor free over 7 weeks were challenged orthotopically in the right posterior mammary fat pad with 0.5 × 10^6^ live 4T1-luc tumor cells. Tumor growth was monitored twice weekly by caliper measurements.

### Tissue harvesting and processing for the analysis of immune cells

Nine days after NPS treatment, mice were euthanized and draining lymph nodes were collected. Draining lymph nodes from tumor bearing mice without NPS treatment were used as control. Single cell suspicions were prepared to analyze immune cells including CD3, CD4, CD8 and tissue-resident marker CD103. To examine effect memory and central memory T cells, animals with tumor free over 3 months after NPS treatment were euthanized. Spleens were harvested. Spleens of tumor bearing mice were used as control. Single cell suspensions were prepared from spleens then stained with CD3, CD4, CD8, CD44 and CD62L antibodies.

To quantify IFN-γ producing T cells, intracellular staining was carried out. Splenocytes (2 × 10^6^/ml) 1 ml per well were incubated with media or plate bound low endotoxin/azide free LEAF anti-CD3 Ab (0.5 µg/mL in DPBS) in a 24-well plate. cells were incubated for 6 h and monensin added for the final 4 h.

### Detection of H_2_O_2_ using the amplex red hydrogen peroxide/peroxidase assay kit

A protocol provided by the manufacturer was modified and adopted to quantify the concentration of H_2_O_2_ released from cells. Briefly, 4T1-luc cells were treated with various parameters of NPS as described above, following which 25,000 cells were seeded into individual wells of a 96-well plate pre-filled with 50 μL cell culture media per well. Amplex Red reagent/HRP working solution, 50 μL, was added to each microplate well and placed in the chamber of PLUOstar Omega fluorescent microplate reader with the Atmospheric Control Unit that enables us to set temperature at 37 °C and CO_2_ at 5%. This setting allowed for measuring the samples continuously at designated time points and with minimal disturbance of cells. The measurement of H_2_O_2_ was done by top-reading with cell adhered to the bottom of the plate wells without mixing. Fluorescence intensity was measured at the excitation of 550 nm and emission of 590 nm. Cell culture medium without cells treated with NPS (100 pulses) was used as the background control. Serial dilutions of H_2_O_2_ (concentrations from 10 to 0.03125 μM) were used to establish a standard curve. In some cases, the fluorescence intensity or relative fluorescence units (RFU) were used to indicate the relative levels of H_2_O_2_.

### Measurement of ROS by DHE

DHE (10 μM) was added into 4T1-luc cell suspensions followed by incubation at 37 °C and 5% of CO_2_ for 15 min. Next, the cells were treated with NPS as described above following which 25,000 cells were then taken out of the NPS cuvette and seeded into individual wells of a 96- well plate pre-filled with 100 μL of cell culture media (without phenol red) per well. Superoxide anion (O_2_¯) converts DHE to ethidium which was monitored by the plate reader over time. The fluorescence intensity was measured as described above with a fluorescence excitation wavelength at 520 nm and emission wavelength at 600 nm. The difference here is that no superoxide standard curve was established, so the relative fluorescence units (RFU) was used as the relative level of ROS.

### Live cell imaging using MitoSOX Red mitochondrial superoxide indicator

Briefly, 4T1-luc cells with or without NPS treatment were placed into a 6-well plate with 0.5 × 10^6^ cells per well. Cells were then incubated at 37 °C, 5% CO_2_ overnight. After gentle removal of culture medium 1.0 ml of working solution containing 5 μM MitoSOX, which was made by a 1:1000 dilution from the 5 mM stock solution in DMSO into culture media, was added into each well and the plate was incubated at 37 °C for 10 min in the dark. The incubation time for MitoSOX loading cells was adopted according to manufacturer’s instructions. The MitoSOX working solution was then removed and replaced with nuclear staining buffer, 0.5 μg/mL Hoechst 33342 in DMEM without phenol red. Cells were imaged immediately under a fluorescence microscope (Olympus BX51). The DAPI filter was used to detect nuclear staining and the TRITC filter was used to detect oxidized MitoSOX Red in the cells.

### Cell viability assay

WST-1 cell viability assay was described previously^55^. Briefly, 10 µL (5 × 10^6^/mL) of cell suspension after exposure to NPS with or without ROS blockers was placed into a clear-flat-bottom 96-well plate filled with 90 µL complete medium per well. All ROS blockers including Trolox (1 mM), RA (100 μM), Vitamin C (0.5 mM), NAC (3 mM) and Sodium pyruvate (10 mM), and with their corresponding concentrations previously screened for their ability to block ROS generation were examined by cell viability assays. Cells were incubated at 37 °C and 5% CO_2_. Following an 18-h incubation 10 μL of WST-1 reagent was added to each well. Cells were incubated with WST-1 for 2 h and then measured by Multiskan MCC/340 microplate reader (Fisher Scientific, Hampton, NH) with a test wavelength of 450 nm and a reference wavelength of 630 nm. Cell viability (%) was calculated using the formula: Treated sample (OD450-OD630)/control (OD450-OD630) × 100. 4T1-luc cells without NPS exposure but otherwise treated the same way as those exposed to NPS were used as the control.

### Generation and activation of bone marrow-derived DCs (BMDCs)

BMDCs were prepared from harvested bone marrow cells by 8 days of culture and differentiation in the presence of 20 ng/ml GM-CSF (R&D). BMDCs (2 × 10^5^) were then incubated with 4T1-luc cells (2 × 10^5^) treated with a lethal dose of NPS (100 pulses, 60 ns, 50 kV/cm and 1 Hz) alone or with the addition of Trolox (1 mM) in a 24-well plate at 37 °C, 5% of CO_2_ for 2 days. BMDCs in the presence of either media alone or LPS (5 μg/mL), but without 4T1-luc cells, were used as negative and positive controls, respectively. Cells were harvested to analyze cell surface activation markers (MHC-I/II, CD40 and CD80) by flow cytometry.

### Vaccination and tumor challenge

Mice were shaved and subcutaneously (SC) inoculated with 3 × 10^6^ NPS treated 4T1-luc cells with or without a ROS blocker in 100 μL sterile saline. Control animals were inoculated SC with the same quantity of cells lysed with 3 freezing/thawing cycles. There were 4 groups: control (Ctrl), NPS treated cells (NPS), NPS treated cells with preincubation with Trolox 1 mM (NPS + Trolox) or sodium pyruvate 10 mM (NPS + SP). All 20 mice (n = 5 per group) were challenged with 0.5 × 10^6^ 4T1-luc live cells in 50 μL sterile saline in the left posterior mammary fat pad 10 days later. Tumor growth was monitored twice weekly by caliper measurements. Animals with complete tumor rejection were followed for at least 4 months, and those with tumor growth were euthanized when the volume of tumor reached 1.5 cm^3^ unless euthanasia was required earlier due to other criteria described for experimental endpoints in the approved IACUC protocol.

### Flow cytometry analysis

2 × 10^5^ BMDCs were incubated with an antibody cocktail (anti-MHC-I/II, CD40 and CD80, each at 1 μg per million cells) in 100 μL FACS buffer (2% FBS DPBS) at room temperature for 30 min. Cells were then washed with 2 mL FACS buffer twice and resuspended in 0.5 mL FACS buffer for flow cytometric analysis by MACSQuant Analyzer 10 (Miltenyi Biotec). Cells stained with isotype antibodies were used as negative controls. All stained cells were run through a flow cytometer. Live cells were gated in a forward scatter (FSC) versus side scatter (SSC) plot then analyzed for cell surface biomarkers.

For intracellular staining, 2 × 10^6^ splenocytes were prepared by pre-incubation with purified anti-CD16/32 (Fc block), followed by surface labeling of cells with anti-CD3 pacific blue, anti-CD4 FITC and anti-CD8 PerCP followed by intracellular staining using mAbs anti-IL-17A PE-Cy7 and anti-IFN-γ PE after fixation and permeabilization with fixation and permeabilization buffer. Samples were analyzed on a flow cytometer (FACSAria, BD Biosciences).

### Statistical analysis

Values were presented as the mean ± standard deviation (SD) or standard error (SE). Student’s *t* test was utilized to compare quantitative data including tissue-resident memory, effector and central memory T cells between two groups. One Way ANOVA (3 or more groups) was utilized to analyze the quantitative data including cell viability and the level of ROS (H_2_O_2_ concentration or RFU). To compare the dynamic change of ROS among different treatment groups, the accumulated ROS or area under curve was calculated and analyzed. If One Way ANOVA showed statistical significance, then Pairwise Multiple Comparison Procedures (Holm-Sidak method) would be done to compare various pairs of groups. Chi-square was employed to analyze the vaccine effect or the rate of protection. If Chi-square for multiple groups showed statistical significance, then Pearson Correction and Chi-square between two groups would be done to compare two groups. Animal survival will be analyzed with Kaplan–Meier Survival LogRank analysis. Statistical significance is assumed at *p* < 0.05. All statistical analysis was completed using SigmaPlot 12.0 (Systat Software, Inc., San Jose, CA).

### Ethical approval

All animal experiments in this study were reported in accordance with ARRIVE guidelines. All 10 Essential requirements were described in the sections of Material & Methods and Results.
